# "Florence Warden" on Sandgate Convalescent Homes

**Published:** 1900-03-10

**Authors:** 


### "FLORENCE WARDEN" ON SANDGATE CONVALESCENT HOMES.

Mrs. Florence James (Florence Warden) writes: To you, as an authority in the matter of health and disease, and one to whom the sanitary condition of England appears in its rightful importance, I address myself on a matter connected with the public health, in the hope that you will be able to give this particular matter some attention in your influential columns. I enclose you a copy of the *Folkestone Herald*, which will give you the particulars of a grave danger which hangs over the little neighbouring town of Sandgate, through the importation, by a man named John James Jones, of batches of diseased persons from London, whom he dumps down, not into specially-constructed dwellings, but into any old house he finds vacant. This man has been for years on the black list of the Charity Organisation Society, and is a rogue of the first water. No sooner is one abuse connected with his hugger-mugger hospitals discovered and rectified than a fresh one starts up. Nothing can be less well adapted than these dwellings for the purposes to which they are now put. The drainage is inadequate; one of the houses is only fit for pulling down; another has no accommodation for cooking, and has all food cooked in another "home" on the opposite side of the street. Children suffering from different contagious diseases are herded together under the same roof; excursionists are entertained in the garden of a house full of diseased and sickly children. Some of these sick children are carried at night, wrapped in blankets, from one house to another. The abuses in connection with the "homes" find an apt illustration in a case the particulars of which are also given in the paper, extracts from which I send. The West Ham Guardians withdrew a large number of children from the care of this Jones and his family on discovering, by a surprise visit, the way in which they were treated. If you will take up this matter, and expose in your valuable paper this disgrace to the sanitary condition of one of our towns, you will be doing a great public service.

[*** We quite agree with our correspondent. The abuses connected with these sham convalescent homes are notorious, and the fact that the man mentioned above is able to go on year after year living upon the pretence of providing proper accommodation for convalescent children is a proof, if proof were needed, of the infinite gullibility of the British public. Anyone who chooses to make an appeal in the name of sick children has the pockets of the charitable almost at his disposal, and in these so-called convalescent homes we have an example of the injury done by such injudicious giving. Instead of being a benefit to the children these homes are mere hotbeds of disease. So great in fact is the mortality among the children that the deaths in these homes more than double the death-rate of Sandgate. According to the annual report of the medical officer of health, as set out in the *Folkestone Herald*, out of the total of 55 deaths which occurred in Sandgate during the year, no less than 31 took place in the various convalescent homes, among the latter being all the deaths from zymotic disease that were registered in the district. Again, these homes are credited with thirteen deaths from phthisis, while if we turn from deaths to notifications it is seen that out of fourteen cases of diphtheria notified to the medical officer of health from the whole district no less than thirteen came from the convalescent homes. We cannot then wonder that the residents in Sandgate raise a protest against such a misuse of their locality as is involved in dumping down these infectious cases in their midst. But putting on one side the feeling of the residents, it is obviously a great scandal that by the unthinking charity of careless givers any man should be allowed for his own purposes to run such ill-regulated, ill-constructed, and ill-managed concerns as these convalescent homes, which, in addition to being a nuisance to the neighbourhood, would appear to be veritable death-traps to the unfortunate children who enter them in the hope of regaining health.

